# Health-led growth hypothesis and health financing systems: an econometric synthesis for OECD countries

**DOI:** 10.3389/fpubh.2024.1437304

**Published:** 2024-07-24

**Authors:** Emre Atilgan, H. Murat Ertuğrul, Onur Baycan, Hakan Ulucan

**Affiliations:** ^1^Department of Health Management, Trakya University, Edirne, Türkiye; ^2^Department of Economics, Anadolu University, Eskişehir, Türkiye

**Keywords:** health-led growth hypothesis, health expenditure, economic growth, OECD countries, health financing systems, second generation panel data analysis

## Abstract

**Introduction:**

This study investigates the Health-Led Growth Hypothesis (HLGH) within OECD countries, examining how health expenditures influence economic growth and the role of different health financing systems in this relationship.

**Methods:**

Utilizing a comprehensive analysis spanning 2000 to 2019 across 38 OECD countries, advanced econometric methodologies were employed. Both second-generation panel data estimators (Dynamic CCEMG, CS-ARDL, AMG) and first-generation models (Panel ARDL with PMG, FMOLS, DOLS) were utilized to test the hypothesis.

**Results:**

The findings confirm the positive impact of health expenditures on economic growth, supporting the HLGH. Significant disparities were observed in the ability of health expenditures to stimulate economic growth across different health financing systems, including the Bismarck, Beveridge, Private Health Insurance, and System in Transition models.

**Discussion:**

This study enriches the ongoing academic dialog by providing an exhaustive analysis of the relationship between health expenditures and economic growth. It offers valuable insights for policymakers on how to optimize health investments to enhance economic development, considering the varying effects of different health financing frameworks.

## 1 Introduction

For over 50 years since Mushkin’s foundational study (1), the link between health expenditure and economic growth has been a crucial topic in academic and policy discussions. This persistent focus highlights the complexity of the relationship between health investments and economic development. Scholars generally agree that health is a vital component of human capital, which is critical for economic growth. As countries strive to provide high-quality healthcare, the connection between health spending and economic growth becomes increasingly important, with significant implications for policy formulation, economic development, and societal well-being.

The health-economic growth nexus refers to the reciprocal relationship between health expenditures and economic growth. This perspective recognizes that health expenditures can influence economic growth through various channels, such as human capital accumulation, labor productivity, and demographic dividends. Simultaneously, economic growth can enable higher health expenditures, as wealthier countries have more resources to invest in healthcare. This nexus suggests a dynamic and interdependent relationship, with feedback effects that can either strengthen or weaken the link between the two variables (2).

The Health-Led Growth Hypothesis (HLGH) posits that investments in health positively impact economic growth. This hypothesis is linked to endogenous growth theory, which emphasizes the importance of human capital accumulation and investment as drivers of economic growth (3–7). The rationale is that healthier populations are more productive and possess higher human capital, leading to greater output, innovation, and adaptability to economic changes. Health expenditure, in this context, is seen as an investment yielding economic returns through improved health outcomes, increased labor productivity, and demographic dividends (1, 8–13).

On the other hand, the wealth hypothesis suggests that economic growth leads to higher health expenditures, as countries with higher GDP *per capita* can allocate more resources to healthcare. This hypothesis indicates that as countries grow wealthier, they can afford to invest more in healthcare, resulting in better health outcomes for their populations (14). Accordingly, economic growth acts as a prerequisite for increased healthcare spending and the improvement of health results (15). The wealth hypothesis is based on the “income elasticity of demand” for healthcare, which indicates that as incomes rise, the demand for healthcare services also increases, leading to higher health expenditures (16). Health care is considered a luxury good, with its demand rising faster than income (17).

The discussion on the interplay between healthcare spending and economic growth is extensive and multifaceted. Various econometric methodologies have been used to study this complex relationship across different national contexts. The literature commonly indicates a favorable association between healthcare expenditure and economic advancement; however, divergences in methodology and geographical disparities result in a spectrum of outcomes, emphasizing the intricate characteristics of this relationship.

Methodologically, there is a division within the academic community, characterized by a significant divergence in the methodologies favored for studying the HLGH in OECD countries. Scholars such as Gerdtham and Lothgren (18), Baltagi and Moscone (19) and Kumar (20) support the use of panel data techniques for capturing broader trends across nations. On the contrary, Atilgan, Kilic and Ertugrul (21) and Tang and Ch’ng (22) advocate for the utilization of time-series methodologies. They argue that these approaches are more appropriate for examining the distinctive attributes of specific countries and questioning the presumption of uniformity that underlies panel data techniques.

Empirical evidence from Newhouse (23) and Gerdtham and Jönsson (17) supports a positive link between GDP *per capita* and health expenditures across various economies, regardless of their development status. This consensus is echoed by Behera and Dash (24) and Beylik, Cirakli, Cetin, Ecevit and Senol (25) who found a positive relationship between healthcare spending and economic growth in Indian states and OECD countries, respectively, by using ARDL models. Additionally, Jakovljevic, Timofeyev, Ranabhat, Fernandes, Teixeira, Rancic and Reshetnikov (26) explored this relationship within G7 and EM7 nations, while Ozyilmaz, Bayraktar, Isik, Toprak, Er, Besel, Aydin, Olgun and Collins (27) found a bidirectional causal relationship in EU countries. However, in MENA countries, the correlation between health expenditure and economic growth is not straightforward, as evidenced by research employing panel OLS, FMOLS, and DOLS approaches (28). Inquiries into the connection between healthcare expenditure and economic growth in developing countries provide a contrast to the focus on OECD nations. This sheds light on the policy implications of healthcare spending in boosting a healthier and more productive population, which in turn could stimulate economic progress (29–32).

The scholarly landscape is enriched by a variety of econometric approaches, from the panel VAR method (33) and Baumol’s model of ‘Unbalanced Growth’ (33) to panel regression analysis (34). This diversity extends to the exploration of the convergence hypothesis (35) and the Driscoll-Kraay approach (25), emphasizing the multifaceted nature of the health expenditure-economic growth paradigm. Time series analysis also plays a significant role in elucidating long-term relationships and expenditure behavior within OECD countries (9, 36, 37).

The intricate relationship between health expenditures and economic growth is influenced by factors such as economic development stages, healthcare system structures, financing configurations, governance quality, and policy efficacy. These elements impact developed and developing nations differently, highlighting the need for ongoing research to understand this relationship fully. Central to this discourse is the role of governance and institutional quality, as underscored by Rodrik, Subramanian and Trebbi (38), who emphasize the critical importance of sound governance and institutional integrity in mediating the health expenditure-economic growth dynamic. This claim is supported by additional studies (39, 40), that emphasize the crucial importance of strong institutions, indicating that efficient governance and institutional excellence play a key role in utilizing health expenditure for economic progress.

Our study aims to empirically analyze the HLGH within the OECD countries. We will examine the health-economic growth nexus across different national settings, considering unique parameter estimates for each country. This analysis will explore how different health financing systems—Bismarck, Beveridge, private insurance, and System in Transition (former Semaschko) models—affect the relationship between health investments and economic outcomes. By categorizing nations based on their health funding frameworks, we hope to provide insights into how these systems influence economic growth. This research is crucial for developing health strategies and fiscal models that promote health and economic prosperity in diverse national contexts.

In our study, we utilize panel data techniques to analyze the complex relationship between health expenditures and economic growth. Panel data techniques are essential for analyzing datasets that combine cross-sectional and time series data, providing a richer analytical framework compared to pure time series or cross-sectional methods. These techniques allow for the control of unobserved heterogeneity, capture dynamic relationships, and improve the efficiency of estimates (41).

In the literature, many studies have utilized panel data techniques to analyze the HLGH. However, these studies predominantly employed first-generation models, which are constrained under certain conditions. First-generation methods, such as the Panel ARDL with the Pooled Mean Group (PMG) estimator, Fully Modified Ordinary Least Squares (FMOLS), and Dynamic Ordinary Least Squares (DOLS), assume cross-sectional independence and homogeneity among the units (42–44). These assumptions often do not hold in real-world datasets, leading to biased estimates in the presence of cross-sectional dependence and heterogeneity. It is noteworthy that previous studies have been limited to first-generation methods, highlighting a significant constraint in panel data approaches.

To address these limitations, our study employs both first-generation and second-generation panel data estimators. First-generation methods provide a baseline for comparison and robustness checks. For a more robust and reliable analysis, we utilize second-generation panel data estimators, including the Dynamic Common Correlated Effects Mean Group (CCEMG), Cross-Sectionally Augmented Autoregressive Distributed Lag (CS-ARDL), and Augmented Mean Group (AMG) estimators. These advanced methods account for cross-sectional dependence and heterogeneous slopes, offering more accurate and comprehensive estimates (45). By employing both generations of panel data estimators, we aim to mitigate the constraints of previous studies and provide a more nuanced understanding of how health investments impact economic growth across different health financing systems. This comprehensive approach allows us to derive more reliable policy implications that can guide the development of effective health strategies and fiscal policies aimed at fostering health and economic prosperity in diverse national contexts.

Our study’s significance lies in its potential to contribute to the academic discussion on the HLGH. By offering new insights into the relationship between health expenditures and economic growth, particularly concerning varied healthcare financing systems, we aim to address gaps in the current literature. The implications of our research extend beyond academic interest, providing essential information for policymakers and stakeholders to enhance the synergy between health expenditure and economic growth, ultimately promoting societal welfare and sustainable progress. Our findings will contribute to the ongoing dialog in this field and set the stage for future research and policy initiatives.

The structure of our study is as follows: after this introduction, we describe the data and methodology used, followed by an empirical analysis of the data. We then discuss the policy implications of our findings and conclude with a summary of the key results and recommendations for future research.

## 2 Data and methodology

As discussed in the introduction section, the relationship between health expenditures and GDP growth is complex and potentially bidirectional. We acknowledge that causality may run from health expenditures to GDP growth or vice versa. However, our main research question specifically focuses on analyzing the effect of health expenditure on GDP growth using a production function within a growth model. While the relationship may indeed be bidirectional, our study aims to maintain a clear focus on this primary objective.

To achieve this, we have adopted an analytical approach that isolates the impact of health expenditures on economic growth, consistent with the HLGH. This focused approach allows us to provide targeted insights and policy recommendations regarding the role of health investments in promoting economic development. Although the bidirectional nature of this relationship warrants further exploration, for the purposes of our current study, we have chosen to prioritize the analysis of the effects of health expenditures on GDP growth.

In our study, HLGH was empirically examined across 38 OECD countries, utilizing an annual dataset spanning from 2000 to 2019. The variables employed within this research encompassed Gross Domestic Product (GDP) *per capita* at constant prices, Gross Capital Formation at constant prices, labor force prices, and *per capita* health expenditures at constant prices. The dataset for these variables was carefully acquired from the World Development Indicators (WDI) database, which is curated by the World Bank (46). Consistent with scholarly conventions, natural logarithms of the variables were computed to facilitate an analysis centered on elasticity values.

We employed both first-generation and second-generation panel data models to analyze the impact of health expenditures on economic growth. First-generation panel data models, such as the Pooled Mean Group (PMG) estimator, Fully Modified Ordinary Least Squares (FMOLS), and Dynamic Ordinary Least Squares (DOLS), are designed under the assumption of cross-sectional independence and homogeneity among units (42–44). These models are effective when such assumptions hold true but may produce biased estimates when there is cross-sectional dependence or heterogeneity in the data. First-generation models typically focus on estimating long-run relationships and cointegration in panel datasets. In contrast, second-generation panel data models, such as the Dynamic Common Correlated Effects Mean Group (CCEMG), Cross-Sectionally Augmented Autoregressive Distributed Lag (CS-ARDL), and Augmented Mean Group (AMG) estimators, account for cross-sectional dependence and heterogeneous slopes (45). These models are more flexible and robust in dealing with complex data structures commonly found in macroeconomic datasets. Second-generation models incorporate common factors and unobserved heterogeneity, making them particularly suitable for analyzing data with cross-sectional dependencies.

First-generation models operate under the presumption that the cross-sectional units in the panel are independent of each other. This means that they do not account for potential correlations between units, which can arise due to shared shocks or common trends. As a result, while these models are suitable for simpler datasets where such independence can be reasonably assumed, they may lead to biased or inefficient estimates in more complex datasets. Furthermore, these models typically assume homogeneity in the slopes of the explanatory variables across different units, which can be a significant limitation when analyzing diverse datasets with varying underlying relationships.

In contrast, second-generation models offer a more advanced approach by explicitly addressing these limitations. These models incorporate mechanisms to account for cross-sectional dependence by including common factors that can capture the shared influences affecting the different units in the panel. This inclusion helps mitigate the biases that arise from ignoring such dependencies. Moreover, second-generation models allow for heterogeneous slopes, which means they can provide more accurate and tailored estimates for each cross-section. This flexibility is crucial when dealing with datasets that encompass a wide variety of units with different characteristics and relationships.

Another key advantage of second-generation models is their ability to handle both short-term and long-term dynamics. For instance, the CS-ARDL model can simultaneously estimate short-term fluctuations and long-term equilibrium relationships, providing a comprehensive view of the interactions between variables over different time horizons. This dual capability is particularly valuable in macroeconomic analyses where short-term and long-term effects often differ significantly. Furthermore, second-generation models are generally better suited for dealing with complex datasets. They are designed to manage the intricacies of macroeconomic data, which often exhibit both cross-sectional dependence and heterogeneity. This makes them more versatile and reliable for a wide range of applications, from policy analysis to forecasting.

In summary, while first-generation panel data models are useful for basic applications with simpler data structures, second-generation models offer significant improvements in terms of flexibility, robustness, and accuracy. By explicitly accounting for cross-sectional dependence and allowing for heterogeneous slopes, second-generation models provide a more nuanced and reliable analysis of complex datasets. This makes them particularly well-suited for our study, which aims to explore the intricate relationship between health expenditures and economic growth across different health financing systems in OECD countries. By including this overview, we aim to provide a clearer understanding of the advantages and limitations of both first- and second-generation panel data models, thereby enhancing the methodological rigor and transparency of our study.

The model presented in Equation (1) has been employed to test the Health-Led Growth Hypothesis.


(1) $${\mathrm{LY}}_{\mathrm{it}}=\lambda_{i}d_{t}+\alpha_{1i}{\mathrm{LGCF}}_{\mathrm{it}}+\alpha_{2i}{\mathrm{LL}}_{\mathrm{it}}+\alpha_{3i}{\mathrm{LH}}_{\mathrm{it}}+u_{\mathrm{it}}$$

$u_{it}=\theta_{i}f_{t}+\varepsilon_{it},$ i=1,2,…,Nandt=1,2,…T

In equation (1), LY is the dependent variable, LGCF, LL and LH are the explanatory variables as described in Table 1. The variables $d_{t}$ and $f_{t}$ correspond to observed and unobserved common effects respectively, reflecting the influences that are both measurable and latent across the dataset. Lastly, $\varepsilon_{it}$ signifies the error term, encapsulating the random variations not explained by the model.

**Table 1:** Variable list.

Variable	Description	Abbreviations
GDP	GDP *per capita*, deflated to 2010 USD	LY
Gross Capital Formation	Gross Capital Formation, deflated with 2010 USD	LGCF
Labor Force	Labor Force	LL
Health Expenditure	Health Expenditure *per Capita*, deflated to 2010 USD	LH

In our applied modeling, we systematically addressed cross-sectional dependency, a critical concern in panel data econometrics, by initially applying the bias-adjusted LM test formulated by Pesaran, Ullah and Yamagata (47). This step is foundational, given that overlooking such dependencies may engender distortions in unit root test results and bias in model estimations, as delineated by O’Connell (48) and Sarafidis and Robertson (49). The assumption of slope homogeneity was critically evaluated using the test by Pesaran and Yamagata (50) and Blomquist and Westerlund (51) given that heterogeneity in slopes is a frequent characteristic in extensive panel data (52, 53). In instances of detected cross-sectional dependency and slope heterogeneity, we incorporated the CIPS unit root test designed by Pesaran (54) to adjust for these dependencies, moving beyond the restrictive assumptions of first-generation panel data models. Upon establishing that the variables were I (1), we assessed the cointegration relationships utilizing Westerlund (55) Durbin Hausman cointegration test, a second-generation test that accounts for cross-sectional dependency.

Subsequent to detecting slope heterogeneity and cross-sectional dependency we applied the second generation panel data models, Dynamic CCEMG (Common Correlated Effects Mean Group) estimator devised by Chudik and Pesaran (45), CS-ARDL (Cross-Sectional Augmented Distributed Lag) estimator and AMG (Augmented Mean Group) estimator. The Dynamic CCEMG estimator accounts for cross-sectional dependence by incorporating common factors into the regression model. This method allows for heterogeneous slopes and intercepts across cross-sections, making it suitable for datasets with diverse country characteristics as mentioned before. The primary effect estimated by CCEMG is the long-term relationship between health expenditures and GDP growth while controlling for unobserved common shocks. The CS-ARDL estimator is designed to handle both short-term and long-term dynamics in the presence of cross-sectional dependence. This method augments the standard ARDL model by including cross-sectional averages of the dependent and independent variables, addressing potential biases from cross-sectional correlations (56). CS-ARDL estimates both the immediate (short-term) effects of health expenditures on economic growth and the long-term equilibrium relationship. The AMG estimator is particularly effective in dealing with heterogeneous slopes and unobserved common factors that might affect the panel data. This method extends the Mean Group (MG) estimator by augmenting it with common correlated effects, allowing for the estimation of both short-term and long-term coefficients while accounting for cross-sectional dependence. AMG provides robust estimates of the impact of health expenditures on GDP growth by addressing potential endogeneity and omitted variable bias (57, 58).

We utilized these three estimators to cross-validate our findings and ensure that our results are not sensitive to the choice of estimation technique. Each estimator has unique strengths: CCEMG excels in capturing long-term relationships under cross-sectional dependence. CS-ARDL provides insights into both short-term and long-term dynamics. AMG addresses unobserved common factors and heterogeneity, enhancing the robustness of our estimates. By applying these estimators, we ensure a comprehensive analysis that robustly supports our conclusions regarding the impact of health expenditures on economic growth across OECD countries.

The Dynamic CCEMG model is as:


(2) $$\begin{array}{l} LY_{\left\{it\right\}}=\alpha_{i}+\beta_{1}LY_{\left\{it-1\right\}}+\beta_{2}LGCF_{\left\{it\right\}}+\beta_{3}LL_{\left\{it\right\}}+\\ \beta_{4}LH_{\left\{it\right\}}+\psi \left(\bar{\left\{LY_{t}\right\}},\bar{\left\{LGCF_{t}\right\}},\bar{\left\{LL_{t}\right\}},\bar{\left\{LH_{t}\right\}}\right)+\varepsilon_{\left\{it\right\}} \end{array}$$

In Equation (2), LY is the dependent, LCGF, LL and LH are the independent variables, for country *i* at time *t*, $\alpha_{i}$ is the country-specific intercept, $\beta_{1}$, $\beta_{2}$, $\beta_{3}$ and $\beta_{4}$ are the coefficients for the lagged dependent variable and independent variables, ψ is a function representing the common correlated effects, which are typically approximated by the cross-sectional averages of the dependent and independent variables (denoted by overbars) and $\varepsilon_{it}$ is the error term.

The CS-ARDL model is as:


(3) $$\begin{array}{l} LY_{it}=\alpha_{i}+\overset{p}{\underset{j=0}{\sum }}\beta_{j}LY_{i,t-j}+\overset{q}{\underset{j=0}{\sum }}\gamma_{1j}LGCF_{i,t-j}+\overset{q}{\underset{j=0}{\sum }}\gamma_{2j}LL_{i,t-j}\\ +\overset{q}{\underset{j=0}{\sum }}\gamma_{3j}LH_{i,t-j}+\lambda_{1}{\bar{LY}}_{t}+\lambda_{2}{\bar{LGCF}}_{t}+\lambda_{3}{\bar{LL}}_{t}+\lambda_{4}{\bar{LH}}_{t}+\varepsilon_{it} \end{array}$$

In Equation (3) LY is the dependent, LCGF, LL and LH are the independent variables, for country *i* at time *t*, $\alpha_{i}$ is the country-specific intercept, $\beta_{j}$ represents the coefficients for the lagged dependent variable (for lags (j=0) to (p)), $\gamma_{1j}$, $\gamma_{2j}$ and $\gamma_{3j}$ are the coefficients for the lagged independent variables, respectively, (for lags (j=0) to (q)), ${\bar{LY}}_{t}$, ${\bar{LGCF}}_{t}$, ${\bar{LL}}_{t}$ and ${\bar{LH}}_{t}$` are the cross-sectional averages of the dependent and independent variables at time *t*, $\lambda_{1}$, $\lambda_{2}$, $\lambda_{3}$ and $\lambda_{4}$ are the coefficients for the cross-sectional averages, capturing the common correlated effects and $\varepsilon_{it}$ is the idiosyncratic error term.

The AMG model is as:


(4) $$\begin{array}{l} LY_{\left\{it\right\}}=\alpha_{i}+\gamma_{t}+\beta_{1i}LY_{\left\{it-1\right\}}+\beta_{2i}LGCF_{\left\{it\right\}}\\ +\beta_{3i}LL_{\left\{it\right\}}+\beta_{4i}LH_{\left\{it\right\}}+\lambda \hat{\left\{dt\right\}}+\varepsilon_{\left\{it\right\}} \end{array}$$

In Equation (4) $\alpha_{i}$ is the country-specific intercept, $\gamma_{t}$ represents the time-specific effects, $\lambda \hat{\left\{dt\right\}}$ represents the coefficient multiplied by the demeaned time trend, where $\hat{dt}$ is the dynamic process capturing the common dynamic process and $\varepsilon_{it}$ is the idiosyncratic error term. After estimating the above model, the mean group estimator for AMG is obtained by averaging the country-specific coefficients as given in Equation (5):


(5) $$\mathrm{AMG}\left(\beta_{j}\right)=\frac{1}{N}\overset{N}{\underset{i=1}{\sum }}{\tilde{\beta }}_{ji}$$

Where $\mathrm{AMG}\left(\beta_{j}\right)$ is the mean group estimate for the parameter $\beta_{j}$, N is the number of cross-sectional units (countries), ${\tilde{\beta }}_{ji}$ represents the estimated coefficient $\beta_{j}$for the (j)−th independent variable for country (i). j=1,...4, stands for the different independent variables in the model.

We performed a stratified analysis using the AMG estimator to investigate the impact of health expenditures (LH) on economic growth across different health financing models. Our approach involved two key steps. First, we calculated country-specific parameter estimates for LH using the AMG estimator. This provided us with insights into the unique impact of health expenditures on economic growth for each country. Second, we grouped the countries according to their health financing systems: Bismarck, Beveridge, Private Health Insurance, and System in Transition. Within each group, we calculated the mean of the country-specific estimates. This stratified analysis allowed us to understand the average effect of health expenditures on economic growth within each type of health financing system.

By estimating country-specific coefficients, we ensured that our analysis accurately reflects the distinct economic contexts and health financing environments of each country. The stratified analysis for health financing systems provided us with a comprehensive view of how different health financing models influence the effectiveness of health expenditures in promoting economic growth, which is a core objective of our research. In our analysis, we focused specifically on reporting the stratified coefficient of LH, as our primary goal is to examine the Health-Led Growth Hypothesis (HLGH). This targeted approach enables us to draw clear and precise conclusions about how health expenditures influence economic growth across different health financing frameworks, thereby enhancing the relevance and applicability of our findings.

We specifically chose the AMG estimator for our stratified analysis and country-specific estimations over other second-generation models due to its unique advantages in handling cross-sectional dependence and heterogeneous slopes. The AMG estimator effectively incorporates common dynamic processes, which is crucial given the shared economic and health shocks among OECD countries. This capability is essential for accurately capturing the interdependencies across countries within our panel data. This heterogeneity is particularly important when analyzing a diverse group of countries with different health financing systems, as it provides a more nuanced understanding of how health expenditures affect economic growth in different contexts. The AMG estimator is also adept at addressing cross-sectional dependence by incorporating common factors into the regression model. This feature is essential for our study, as it helps mitigate biases that could arise from ignoring such dependencies. By including these common factors, the AMG estimator ensures that the estimated effects are not distorted by unobserved common shocks that affect all countries in the panel. Other second-generation models, while effective, do not offer the same level of flexibility in capturing country-specific effects and managing cross-sectional dependencies simultaneously.

To validate the robustness of our results obtained from the Dynamic CCEMG, CS-ARDL and AMG models, we performed parallel estimations using the conventional panel data models, Panel ARDL model employing the PMG estimator by Pesaran, Shin and Smith (42), as well as the FMOLS and DOLS models, which address concerns of serial correlation and potential endogeneity (44). These models were chosen for their ability to manage the issues identified in our methodological examination, ensuring that our analysis remains coherent, and the conclusions drawn are firmly grounded in empirical evidence.

FMOLS, developed by Phillips and Hansen (43) is designed to provide asymptotically efficient estimates of cointegrating vectors by adjusting for serial correlation and endogeneity. This method modifies the traditional Ordinary Least Squares (OLS) estimator through a series of non-parametric adjustments based on the long-run covariance matrix of the error terms. FMOLS corrects for serial correlation in the residuals using a non-parametric approach and handles endogeneity by modifying the OLS estimator, utilizing corrections derived from the long-run covariance matrix. It involves the use of kernel estimators to adjust the long-run covariance matrix and, as a non-parametric method, avoids assumptions about the specific form of the error distribution. Consequently, FMOLS produces consistent and efficient estimators for cointegration relationships, making it suitable for large samples with complex error structures.

On the other hand, DOLS, introduced by Stock and Watson (44), offers a different approach by augmenting the cointegrating regression with leads and lags of the first differences of the regressors. This parametric method aims to provide consistent estimates of the cointegration vector, directly addressing endogeneity and serial correlation through dynamic adjustments. DOLS adds leads and lags of the differenced independent variables to the cointegrating regression, directly addressing serial correlation by incorporating dynamic terms into the model. It mitigates endogeneity by including leads and lags, reducing the correlation between the error term and the independent variables. As a parametric approach, DOLS relies on the inclusion of specific dynamic terms, making it straightforward to implement. This methodology is often preferred in small samples due to its parametric nature and ease of implementation.

Both FMOLS and DOLS aim to provide robust estimates of cointegrating relationships in the presence of endogeneity and serial correlation, ensuring asymptotically efficient estimates under specific conditions. Despite these similarities, notable differences exist between the two methodologies. FMOLS employs non-parametric corrections based on the long-run covariance matrix, involving more complex adjustments suitable for larger samples. In contrast, DOLS uses parametric corrections by incorporating leads and lags of the differenced regressors, offering a more straightforward implementation, particularly effective in small samples. By employing both DOLS and FMOLS, we ensure that the long-term relationships between health expenditures and economic growth are accurately estimated, taking into consideration the dynamic and potentially endogenous nature of the data. This dual approach enhances the robustness and reliability of our findings, providing a comprehensive understanding of the health-economic growth nexus in OECD countries.

The Panel ARDL approach is used to estimate the relationships between the dependent variable (GDP) and its lags, along with other independent variables and their lags. The model is specified as:


(6) $$\begin{array}{l} LY_{i,t}=\alpha +\overset{p}{\underset{j=1}{\sum }}\phi_{j}LY_{i,t-j}+\overset{q}{\underset{j=0}{\sum }}\beta_{1j}LGCF_{i,t-j}\\ +\overset{q}{\underset{j=0}{\sum }}\beta_{2j}LL_{i,t-j}+\overset{q}{\underset{j=0}{\sum }}\beta_{3j}LH_{i,t-j}+\varepsilon_{i,t} \end{array}$$

In Equation (6) the variables represent the logarithmic transformations of the actual data values to stabilize variance and improve the model’s interpretability.

The DOLS model in the panel data setting is expressed as Equation (7):


(7) $$\begin{array}{l} LY_{i,t}=\alpha_{i}+\beta_{1}\mathrm{LGC}F_{i,t}+\beta_{2}LL_{i,t}+\beta_{3}LH_{i,t}+\overset{k}{\underset{j=-k}{\sum }}\delta_{1j}\\ \Delta LGCF_{i,t-j}+\overset{k}{\underset{j=-k}{\sum }}\delta_{2j}\Delta LL_{i,t-j}+\overset{k}{\underset{j=-k}{\sum }}\delta_{3j}\Delta LH_{i,t-j}+\varepsilon_{i,t} \end{array}$$

Fully Modified OLS (FMOLS) provides an estimator of cointegrating relationships among non-stationary panel data, correcting for both serial correlation and potential endogeneity. The FMOLS formula is specified as Equation (8):


(8) $${\mathrm{LY}}_{i,t}=\alpha_{i}+\beta_{1}{\mathrm{LGCF}}_{i,t}+\beta_{2}{\mathrm{LL}}_{i,t}+\beta_{3}{\mathrm{LH}}_{i,t}+\varepsilon_{i,t}$$

In employing these methods, appropriate diagnostic tests, including stationarity tests, cointegration tests, and post-estimation diagnostics, are performed to ensure the robustness and reliability of the estimated models.

## 3 Results

We used STATA 17 for performing the tests and estimation of the models. The descriptive statistics of the variables is presented in Table 2.

**Table 2:** Descriptive statistics.

Variable	n	Mean	Std. Dev.	Min	Max
LY	760	10,174	0,741	8,077	11,725
LGCF	760	25,052	1,592	21,372	28,989
LL	760	15,636	1,488	12,040	18,934
LH	760	7,834	0,621	6,227	9,139

Our research commenced by analyzing the presence of cross-sectional dependence. This was evaluated using the bias-adjusted Lagrange Multiplier (LM) test for cross-sectional dependence (47). The findings pertaining to the cross-sectional dependence are tabulated in Table 3.

**Table 3:** Cross-sectional dependence test results.

	Value
LM Test	1842*
CD Test	28.05*
Bias Adjusted LM Test	58.59*

*Denote statistical significance at the 1% level. The null hypothesis is no cross-sectional dependence.

For assessing cross-sectional dependency, the null hypothesis posited in the trio of applied tests postulated the absence of such dependency. As evident from the data in Table 3, this initial hypothesis is refuted across all tests, indicating the presence of cross-sectional dependency within the data set.

Slope homogeneity tests are crucial in the realm of econometric analysis, particularly in the study of panel data and linear panel models. These tests are designed to ascertain whether the relationships between variables remain consistent across various units or groups within a dataset. The assumption of slope homogeneity, a cornerstone of first-generation panel data models, if invalidated, could introduce bias into conventional panel data estimators. Consequently, following the cross-sectional dependency analysis, this research undertook an examination of slope heterogeneity within the model. For this purpose, the slope heterogeneity tests formulated by Pesaran and Yamagata (50) and Blomquist and Westerlund (51) were utilized.

Table 4 presents results from the Pesaran and Yamagata (50), and Blomquist and Westerlund (51) slope homogeneity tests, with both tests revealing significant test statistics at the 1% level. This statistical significance suggests a rejection of the null hypothesis that slope coefficients are homogeneous across the panel data. The presence of adjusted statistics further supports this finding, indicating slope heterogeneity, which implies that different units in the panel exhibit varying slope coefficients. This result is critical for model specification and interpretation within the associated study.

**Table 4:** Slope homogeneity test results.

	Pesaran – Yamagata	Blomquist – Westerlund
Δ	21.951*	16.281*
$\Delta_{adj}$	25.346*	18.800*

*Denote statistical significance at the 1% level. In both tests the null hypothesis is slope coefficients are homogenous.

Subsequently, acknowledging the confirmed cross-sectional dependency and slope heterogeneity, the study progressed to employ the Cross-sectional unit root test (CIPS) (54). The outcomes of this unit root analysis are comprehensively detailed in Table 5.

**Table 5:** CIPS unit root test results.

Variable	Level	First-difference
LY	−1.544	−3.283*
LGCF	−1.751	−3.192*
LL	−2.006	−3.495**
LH	−1.870	−3.505**

*, **Denote statistical significance at the 5 and 1% levels, respectively. The null hypothesis is non-stationarity.

The CIPS Unit Root Test results indicate that the variables LY, LGCF, LL, and LH are non-stationary at their levels as their test statistics are not significant. However, once differenced, all variables exhibit stationarity: LY and LGCF at the 5% significance level, and LL and LH at the 1% significance level. Upon consideration of the cross-sectional dependence present within the dataset, the decision has been made to rely on the outcomes of the CIPS test. Consequently, it is assumed that all series exhibit integration of order one, denoted as I (1).

Given that the series were determined to be integrated of order one I (1), the exploration of the cointegration relationship among the series was undertaken utilizing the Durbin–Hausman cointegration test, as formulated by Westerlund (55), which is chosen for its robust accommodation of cross-sectional dependence. This test affords a more sophisticated analysis of cointegration relationships, thereby enhancing the integrity of the results. The Westerlund Durbin–Hausman test’s ability to account for cross-sectional dependence is essential in ensuring that the long-run equilibria inferred from the time series data are not spurious but indicative of a genuine cointegration relationship. The outcomes of the Westerlund Durbin–Hausman cointegration test are presented in Table 6.

**Table 6:** Westerlund Durbin–Hausman cointegration test result.

	Value
Variance ratio	−1.6845**

**Denote statistical significance at the 5% level. The null hypothesis is no cointegration.

According to the Westerlund cointegration test results, the Variance Ratio statistic is found as −1.6845 with a *p*-value of 0.0460. This p-value, being below the conventional significance threshold of 0.05, provides a statistically significant basis to reject the null hypothesis of no cointegration at the 5% level. Hence, the data reveal a long-term equilibrium relationship among the variables within certain panels, suggesting that these non-stationary time series variables are cointegrated and move in tandem over time.

Upon the results of cross-sectional dependence, slope homogeneity, unit root, cointegration tests, it was ascertained that the series exhibit cross-sectionally dependent errors, slope heterogeneity, presence of a unit root and cointegration. The subsequent discourse will revolve around potential estimators, in order to obtain long-run cointegration coefficients, that demonstrate robustness to cross-sectional dependence and/or slope heterogeneity. We employed advanced econometric models including Dynamic CCEMG, CS-ARDL and AMG to rigorously test the HLGH. To substantiate the robustness and reliability of the findings derived from these models, we conducted conventional estimations utilizing the Panel ARDL approach by adopting the PMG estimator. The estimated outcomes derived from the models are delineated in Tables 7, 8.

**Table 7:** Second-generation panel data estimation results.

Variable	CCEMG	CS-ARDL	AMG
LGCF	0.6316*** (0.1748)	0.5915*** (0.0599)	0.5159*** (0.0318)
LL	2.6963** (1.3500)	−0.3440*** (0.1162)	−0.01851*** (0.0732)
LH	0.6000** (0.2916)	0.3744*** (0.1220)	0.2261*** (0.0552)
The HLGL Holds	Yes	Yes	Yes

*, **, ***Denote statistical significance at the 10, 5 and 1% levels, respectively.

**Table 8:** Robustness check: conventional panel data estimation results.

Variable	Panel ARDL	DOLS	FMOLS
LGCF	0.7476*** (0.0223)	0.7056*** (0.0211)	0.7130*** (0.0249)
LL	−0.1827** (0.0811)	−0.6179*** (0.0469)	−0.4651*** (0.0051)
LH	0.1021*** (0.0304)	0.2228*** (0.0158)	0.3556*** (0.0067)
The HLGL Holds	Yes	Yes	Yes

*, **, ***Denote statistical significance at the 10, 5 and 1% levels, respectively.

The empirical investigation into the HLGH across OECD countries yielded substantive evidence, as encapsulated in the estimations presented in Table 7. When applying Dynamic CCEMG, CS-ARDL and AMG econometric methodologies, we discerned a consistent and statistically significant relationship between health expenditure *per capita* and GDP *per capita*, providing robust support for the HLGH. LGCF exerted a positive influence on economic output, with significant coefficients at the 1% level across all models: 0.631 (CCEMG), 0.591 (CS-ARDL), and 0.515 (AMG). This underscores the assertion that capital investment is a pivotal component of economic growth. LL variable revealed a complex pattern, with a significantly positive impact in the CCEMG model at the 5% level (2.696), contrasted by a significant negative influence in the CS-ARDL and AMG models at the 1% level (−0.3440 and − 0.0185, respectively). This complexity may reflect the nuanced effects of labor force size on *per capita* economic output, contingent upon the diverse economic landscapes and labor market dynamics within the OECD countries.

The difference in parameter estimates across various models, particularly when they exhibit different signs, can be attributed to the underlying assumptions and methodological approaches of each estimator. Dynamic CCEMG estimator accounts for cross-sectional dependence by incorporating common factors into the regression model, allowing for heterogeneous slopes and intercepts. This flexibility can lead to more precise estimates but also to differences in parameter signs if common factors influence countries differently (59). The CS-ARDL model captures both short-term and long-term dynamics by including cross-sectional averages of the dependent and independent variables (45). This approach can result in different parameter estimates due to its focus on dynamic relationships and adjustment processes. The AMG (Augmented Mean Group) estimator builds upon the Mean Group (MG) approach by incorporating common correlated effects to address cross-sectional dependence and unobserved heterogeneity. This extension allows the AMG estimator to provide different parameter estimates, especially when significant unobserved common factors are present. By accounting for these common factors, the AMG estimator offers a more robust analysis in panel data models with heterogeneous slopes and weak cross-sectional dependence of the errors (60).

LH maintained a positive and statistically significant effect on GDP *per capita* at the 5% level in the CCEMG model (0.600) and at the 1% level in both the CS-ARDL (0.374) and AMG (0.226) models, signifying the vitality of health investment as an engine for economic advancement. The consistency in health expenditure’s impact across varied analytical frameworks emphasizes its indispensable role in fostering economic development. The findings conclusively affirm the HLGH for the OECD countries.

While our study spans a diverse set of OECD countries, we acknowledge the inherent variability in structural and economic contexts across these nations. This diversity is a critical factor that our methodology addresses using second-generation panel data models. These models provide a nuanced understanding of the health expenditure-economic growth nexus by accounting for country-specific characteristics. Consequently, our findings offer valuable insights that are both generalizable and context-specific, enhancing their relevance for policymaking across different OECD countries. Even though these second-generation models capture the differences among countries in terms of structural and economic contexts, there may still be other factors not captured by our model specifications. This limitation is important to consider when generalizing our results.

After performing the second-generation models, which effectively address cross-sectional dependency and slope heterogeneity, we also employed first-generation panel data models, namely Fully Modified Ordinary Least Squares (FMOLS), Dynamic Ordinary Least Squares (DOLS), and the Pooled Mean Group (PMG) estimator. Even though the results from the second-generation models indicate the presence of cross-sectional dependency and slope heterogeneity, we included first-generation models for several reasons.

First, incorporating first-generation estimators provides a benchmark for comparing the results obtained from second-generation methods. By presenting these estimates, we can illustrate the differences and potential biases that arise when cross-sectional dependency and heterogeneity are not accounted for. This comparative analysis is essential for understanding the extent to which more advanced estimators improve the accuracy and reliability of our findings. Additionally, including first-generation estimations helps validate the robustness of our findings. If the results from both first-generation and second-generation estimators are consistent, it strengthens the credibility of our conclusions by demonstrating that our findings are not sensitive to the choice of estimation technique. Conversely, significant differences between the two sets of results highlight the importance of using advanced techniques to obtain reliable estimates. This approach ensures that our analysis is thorough and that our conclusions are well-supported by multiple methodological perspectives. By employing both first- and second-generation estimators, we aim to provide a comprehensive and robust analysis, demonstrating the improvements and necessity of advanced methods while ensuring that our findings are reliable and credible across different estimation techniques.

Table 8 provides a coherent and compelling set of results from first-generation panel data methodologies. LGCF exhibits a consistently positive and statistically significant effect on the GDP *per capita* across all three methods, with coefficients of 0.7476 (Panel ARDL), 0.7056 (DOLS), and 0.7130 (FMOLS), all significant at the 1% level. This reinforces the notion that investments in the form of capital formation are a pivotal factor in promoting economic prosperity. LL presents an intriguing case with its negative association with GDP *per capita*, significant at the 5% level in the Panel ARDL (−0.1827) and at the 1% level in both DOLS (−0.6179) and FMOLS (−0.4651) estimations. This counterintuitive finding may suggest that an increasing labor force without concurrent increases in job creation or productivity could potentially dilute GDP *per capita*. It might also reflect structural issues within the labor market, such as underemployment or a mismatch between the skills of the labor force and the needs of the economy.

The differences between first- and second-generation panel data models are evident in the parameter estimates for key variables such as gross capital formation (LGCF) and health expenditure (LH). First-generation models, such as FMOLS, DOLS, and PMG, generally produce larger estimates for LGCF and smaller estimates for LH compared to second-generation models. This can be attributed to the assumptions of cross-sectional independence and homogeneity inherent in these models. These assumptions can lead to biased estimates when there is cross-sectional dependence and heterogeneity in the data. Moreover, first-generation models tend to have smaller standard errors due to their parametric nature and the specific handling of dynamic relationships.

Second-generation models, such as Dynamic CCEMG, CS-ARDL, and AMG, account for cross-sectional dependence and heterogeneous slopes, providing more robust and reliable estimates. These models typically yield smaller estimates for LGCF and larger estimates for LH, reflecting a more nuanced understanding of the underlying relationships. The standard errors are generally larger in second-generation models, reflecting the added complexity and flexibility in the estimation process. Second-generation models incorporate cross-sectional dependence by including common factors or averages, which helps to mitigate biases that may inflate estimates in first-generation models. On the other hand, the ability of second-generation models to allow for heterogeneous slopes results in more accurate and representative estimates for variables like LH, which may vary significantly across different countries and contexts. Second-generation models, particularly CS-ARDL, capture both short-term and long-term dynamics, offering a comprehensive view of the relationships between health expenditures and economic growth. This contrasts with the more static approach of first-generation models. The standard errors in second-generation models are larger due to the robust corrections for endogeneity and cross-sectional dependence, providing a more conservative and reliable estimate.

The robustness of the HLGH is further substantiated through the estimations presented in Table 8. The findings of these estimations further corroborate the HLGH in the context of the 38 OECD countries under study. Health Expenditure *per Capita*, the independent variable of particular interest, shows a positive relationship with GDP *per capita* across all models, with coefficients of 0.1021 (Panel ARDL), 0.2228 (DOLS), and 0.3556 (FMOLS), all significant at the 1% level.

The findings of this study indicate that expenditures on health care do not merely lead to enhanced health outcomes but also substantially contribute to economic growth. This highlights the critical value of health as a form of human capital investment. The positive influence of health expenditure on economic growth is particularly compelling, given its consistency across different estimators and robustness checks. It is a finding that holds profound implications for policy formulation, emphasizing the significance of health sector investments in the broader economic agenda of the OECD countries.

In our scholarly endeavor, one of our primary objectives was to meticulously analyze the impact of health financing systems on the HLGH. Understanding how different systems either facilitate or impede the translation of health expenditures into economic growth is crucial for crafting effective health and economic policies. To achieve this, we estimated country-specific coefficients using the AMG estimator, categorizing the countries by their respective health financing models health financing models—namely the Bismarck Model, Beveridge Model, Private Health Insurance Model, and System in Transition (formerly Semaschko) model. These models represent different approaches to delivering and financing health care: The Bismarck model typically involves health insurance funded by employers and employees through payroll deduction, the Beveridge model is characterized by financing through taxation and the health care being provided by the government, the Private Health Insurance model relies heavily on private health insurance as the principal means of covering health costs and the System in Transition country model introduces a unique group, unlike other categories that are often defined by their source of financing, this classification congregates the transitioning economies of Central Europe which characterized by the ongoing transformation of their health systems, indicating a shift from previous models toward new, more market-oriented or mixed systems of healthcare provision and financing (61, 62). This stratified analysis, as presented in our results, allowed us to not only quantify the effect of health expenditure on GDP *per capita* within each country but also to draw comparisons across differing systemic frameworks. The AMG estimator was chosen for estimating country-specific coefficients to effectively manage data heterogeneity and cross-sectional dependence in the global dataset, which also handles unobserved dynamic factors, ensuring robust assessment of the long-term link between health spending and economic growth in each country.

Table 9 presents the country-specific parameter estimations of LH, elucidating the impact of health expenditure on economic growth. We employed the classification framework delineated by Torbica, Fornaro, Tarricone and Drummond (61) for categorizing countries by their health financing mechanisms in the table.

**Table 9:** Health financing systems and country-specific results of the AMG estimator.

Bismarck model	Coeff.	Beveridge model	Coeff.	Private health insurance model	Coeff.	System in transition (former Semaschko model)	Coeff.
Austria	0.131	Australia	−0.581**	Mexico	−0.211	Czechia	0.366***
Belgium	0.396***	Canada	0.108	Chile	0.203	Estonia	0.569***
Colombia	−0.33	Denmark	−0.022	United States	0.128***	Hungary	0.487***
Costa Rica	0.279	Finland	0,019			Poland	0.466***
France	−0.331*	Greece	0.572***			Slovak Republic	0.259***
Germany	0.083	Iceland	0.28**			Slovenia	0.607***
Israel	−0.295	Ireland	0.076				
Japan	0.063*	Italy	−0.057				
Korea, Rep.	−0.159	Latvia	0.733***				
Lithuania	0.727***	New Zealand	0.663**				
Luxembourg	0.381***	Norway	0.112				
Netherlands	−0.066	Portugal	0.591***				
Switzerland	0.885**	Spain	0.394***				
Turkiye	0.596***	Sweden	0.176***				
		United Kingdom	0.292**				
Mean	0.169	Mean	0.224	Mean	0,040	Mean	0.459
Median	0.107	Median	0.176	Median	0,128	Median	0.477
Std. Dev	0.393	Std. Dev	0.341	Std. Dev	0,221	Std. Dev	0.129

*, **, *** Denote statistical significance at the 10, 5 and 1% levels, respectively.

Within the Bismarck Model countries, there is considerable heterogeneity in how health expenditures relate to economic growth. Nations like Belgium, Lithuania, Luxembourg, Switzerland, and Turkiye demonstrate a robust and positive association, while France and Colombia’s negative coefficients suggest a different scenario where health spending does not translate into economic growth. Several countries show positive but not statistically significant results, indicating a potential trend that requires further exploration. The standard deviation within this group is the highest among the health financing models at 0.393, indicating a substantial dispersion in the coefficients and suggesting that the relationship between health spending and economic growth is complex and likely influenced by multiple country-specific factors.

The Beveridge Model countries predominantly show positive coefficients, with several countries like Greece, Latvia, New Zealand, Portugal, Spain, Sweden, and the United Kingdom indicating a statistically significant positive relationship between health expenditures and GDP growth. These findings suggest that, in many cases, the government’s role in financing healthcare is associated with beneficial economic outcomes. However, the case of Australia demonstrates a significant negative impact, highlighting that the relationship between health spending and economic growth can vary greatly even within a similar healthcare financing framework. The standard deviation for the Beveridge Model group is 0.341, which is relatively high but less than the Bismarck Model group, indicating some degree of variability in the impact of health expenditures on economic growth.

The countries operating under the Private Health Insurance Model demonstrate a more varied impact of health expenditures on GDP growth compared to the Beveridge or Bismarck models. The United States shows a clear positive and significant impact, while Mexico and Chile exhibit coefficients that are not statistically significant, with Mexico showing a negative coefficient and Chile a positive one.

The countries transitioning from the Semaschko model demonstrate a uniformly positive and statistically significant impact of health expenditures on GDP growth, which is quite remarkable. The strong coefficients across these countries suggest that the reforms and restructuring of their healthcare systems have been conducive to leveraging health expenditures for economic growth. The transition appears to be associated with improved efficiency and effectiveness in health spending. The standard deviation for this group is relatively low (0.129), indicating a consistent pattern of positive economic outcomes from health expenditures across these countries. This consistency might be due to the focused efforts and reforms undertaken by these countries to improve their healthcare systems during the transition phase.

Across all models, the mean coefficients are positive, indicating that health expenditure *per capita* generally has a favorable impact on GDP *per capita* across different health financing systems. Yet, the variability within and between these models suggests that the efficiency and effectiveness of health expenditure may be influenced by the particular health financing system in place. The significant coefficients in many countries reinforce the notion that health expenditure is a key factor in economic growth, supporting the HLGH. Moreover, the negative coefficients in some nations indicate that increased health spending alone does not guarantee higher economic output, highlighting the importance of efficiency and the alignment of health expenditures with broader economic policies and objectives.

## 4 Discussion

As the world continues to confront challenges such as aging populations, escalating healthcare costs, and the growing prevalence of chronic illnesses, comprehending the relationship between health spending and economic development is increasingly crucial. In our empirical analysis, health expenditure *per capita* delivered a uniformly positive and significant influence on GDP *per capita*, decisively affirming the HLGH across all econometric estimators. Our findings strengthen the argument for health expenditure as a pivotal component of human capital investment which is crucial for economic advancement. This study’s affirmation of the HLGH holds profound implications for health policy and planning within OECD nations. The empirical evidence that health spending can boost economic growth provides a compelling rationale for investing in robust, efficient healthcare systems. It positions health spending as part of a value-creation strategy rather than just a cost center.

In this research, we also sought to examine the mechanisms through which health systems can facilitate economic prosperity. Our findings indicate that no single health system model consistently surpasses others in performance. Within the Bismarck and Beveridge models, there is a notable variation among countries, highlighting the role of national-specific elements. The Private Health Insurance Model shows less promise for economic benefits arising from health expenditures, albeit with variable outcomes. In contrast, the System in Transition demonstrates a consistently positive effect. The heterogeneity observed across health financing models suggests that merely increasing health expenditures in a vacuum may not guarantee economic dividends. These insights suggest possible avenues for improving the alignment between health system frameworks and overarching economic policy objectives.

The variability observed in the outcomes of the Bismarck model suggests that while payroll-based insurance provides a stable funding mechanism, there is potential for improvement through reforms aimed at increasing flexibility and enhancing strategic purchasing capabilities (63). It is imperative that the structure of contribution rates and the composition of benefits packages are meticulously designed to strike a harmonious balance between the principles of equity and efficiency. The efficacy of the Beveridge model, as evidenced in numerous countries, highlights the significant role that tax-funded healthcare systems can play in fostering economic growth through judicious allocation of resources and ensuring universal health coverage. The implementation of universal coverage is pivotal within the ambit of economic growth and sustainable development initiatives, chiefly because of its profound influence on augmenting human capital through enhanced health outcomes. It serves as a bulwark against the economic ruin caused by steep out-of-pocket healthcare costs, underpins economic productivity by sustaining a workforce that is both healthy and capable, and necessitates the convergence of efforts across various sectors and the determination of political leadership to drive systemic reforms. These reforms are crucial for guaranteeing equitable access to high-quality healthcare services for the entire populace (64). Within the framework of the private insurance model, HLGH was found to be statistically significant exclusively for the United States. In contrast, for other countries within this category, despite significant reforms, initiatives and policy interventions undertaken in Mexico (65, 66) and Chile (67) during the period from 2000 to 2018, our analysis did not identify a statistically significant link between health expenditure and economic growth. This lack of significant correlation may be attributed to the challenges these countries face in adapting to demographic expansion and fluctuations in inflation, which have adversely impacted *per capita* health expenditure, as reported by the OECD (68). The enduring positive influence of health expenditures on economic growth in Post Semaschko Model countries can be attributed to the strategic implementation of health system reforms within these nations (62, 69). Early initiation of comprehensive health reforms is recognized as a key determinant in achieving superior outcomes within their health systems (69).

This study contributes significantly to the academic literature and policy discussions surrounding the relationship between health and economic growth. By empirically supporting the HLGH and providing insights into the structure of health systems, this research lays a groundwork for further academic exploration. The findings highlight the importance of health expenditure as a high-yield investment for policymakers, emphasizing that such spending can substantially benefit economic growth. However, the impact’s magnitude is heavily influenced by the health system’s design and efficiency, as well as the financing methods employed. A deeper analysis of the specific features of each health financing model that account for these differences could provide valuable direction for improving health policies and developing more robust strategies for economic growth.

Despite these methodological precautions, the study acknowledges that its findings are subject to the limitations inherent in econometric modeling. These include the possibility of measurement errors in the variables used, the challenge of fully capturing the complex relationship between health expenditures and economic growth, and the potential for unobserved variables to influence the results. Moreover, the study’s reliance on available data and the assumptions underlying the econometric models may also limit the generalizability of its conclusions.

This study employs advanced econometric techniques to explore the Health-Led Growth Hypothesis across OECD countries. However, several methodological limitations and assumptions need to be acknowledged. Firstly, while the use of second-generation panel data models addresses cross-sectional dependence and heterogeneity, the accuracy of these models depends on the quality and completeness of the available data. Any measurement errors in the data could affect the results. Secondly, the assumption of homogeneity within health financing models may not fully capture the complex and multifaceted nature of health systems in different countries. Although our models account for heterogeneity, there may still be unobserved factors influencing the relationship between health expenditures and economic growth. Thirdly, the study period (2000–2019) does not account for more recent global events such as the COVID-19 pandemic, which could have significant impacts on health expenditures and economic growth. Future research should consider these recent developments to provide updated insights. Lastly, the potential for reverse causality, where economic growth influences health expenditures, is acknowledged. While our primary focus is on the effect of health expenditures on economic growth, the bidirectional nature of this relationship warrants further investigation. By outlining these limitations and assumptions, we aim to provide a clearer understanding of the context in which our results are applicable and the scope for future research.

## 5 Conclusion

This study provides important empirical evidence on the relationship between health expenditures and economic growth across OECD countries. The econometric analysis utilizing advanced panel data estimation techniques consistently affirms the HLGH, demonstrating that health spending exerts a statistically significant positive impact on GDP *per capita*. The results highlight that health expenditures should not merely be viewed from the lens of achieving health outcomes but also as a critical form of human capital investment that can stimulate economic development. The study reveals health spending as an engine of economic growth rather than just a byproduct of growing income levels. Our analysis of country-specific effects reveals nuances in how different health financing systems influence the efficiency of health expenditures in boosting economic output.

## Data availability statement

Publicly available datasets were analyzed in this study. This data can be found at: https://databank.worldbank.org/source/world-development-indicators.

## Author contributions

EA: Conceptualization, Formal analysis, Methodology, Supervision, Writing – original draft, Writing – review & editing. HE: Formal analysis, Methodology, Writing – original draft, Writing – review & editing. OB: Data curation, Funding acquisition, Writing – original draft, Writing – review & editing. HU: Conceptualization, Data curation, Writing – original draft, Writing – review & editing.
